# Impact of Angiotensin I Converting Enzyme Insertion/Deletion Polymorphisms on Dilated Cardiomyopathy and Hypertrophic Cardiomyopathy Risk

**DOI:** 10.1371/journal.pone.0063309

**Published:** 2013-05-14

**Authors:** Jianmin Yang, Yunhan Zhao, Panpan Hao, Xiao Meng, Mei Dong, Ying Wang, Yun Zhang, Cheng Zhang

**Affiliations:** 1 The Key Laboratory of Cardiovascular Remodeling and Function Research, Chinese Ministry of Education and Chinese Ministry of Health, Qilu Hospital, Shandong University, Jinan, Shandong, P.R. China; 2 Shandong University School of Medicine, Jinan, Shandong, P.R. China; University of Tampere, Finland

## Abstract

**Background:**

Genetic factors in the pathogenesis of cardiomyopathies have received a lot attention during the past two decades. Angiotensin I converting enzyme (ACE) insertion/deletion (I/D) polymorphisms were found to be associated with cardiomyopathies. However, the previous results were inconsistent. The current meta-analysis aims to examine the association of ACE I/D polymorphisms and dilated cardiomyopathy (DCM) or hypertrophic cardiomyopathy (HCM).

**Methods:**

Eight studies on DCM (1387 controls and 977 patients) and eight studies on HCM (1055 controls and 827 patients) were included in this meta-analysis.

**Results:**

The overall data showed no significant association between ACE I/D polymorphism and DCM risk. Further subgroup analysis by ethnicity also did not find a significantly increased risk for D allele carriers among East Asians and Europeans. However, the overall analysis suggested that the D allele carriers might be associated with increased risk of HCM (DD/ID vs. II: OR = 1.69, 95% CI 1.04–2.74, *P* = 0.03).

**Conclusion:**

In summary, the meta-analysis indicated that certain ACE I/D polymorphism might be associated with HCM but not DCM susceptibility. Given the limited sample sizes, further large multicenter case-control investigation is needed.

## Introduction

Dilated cardiomyopathy (DCM), which is characterized by ventricular chamber enlargement and systolic dysfunction with normal left ventricular wall thickness, leads to progressive heart failure, arrhythmias, and sudden or heart failure related death. Previous family studies revealed that 20% to 50% of idiopathic DCM had a familial origin, suggesting genetic factors might play an important role in the disease pathogenesis. [1] On the other side, hypertrophic cardiomyopathy (HCM), which is diagnosed in the presence of left ventricular hypetrophy, is also reported to be genetically heterogenous. [2] During the past two decades, several genetic mutations were reported to cause DCM or HCM.

Several genes encoding the components of the renin-angiotensin-aldosterone system (RAAS) have been revealed to be associated with cardiovascular diseases, including hypertension, myocardial infarction, ischemic stroke, and cardiomyopathy. [3], [4], [5] As DCM to be considered, the insertion/deletion polymorphism in the angiotensin I converting enzyme gene (ACE I/D) has been commonly reported. [6], [7] However, the previous results were inconsistent. Raynolds MV et al found that compared with the DD frequency in the control population, the frequency of the ACE DD genotype was 48% higher in individuals with idiopathic DCM. [6] However, Montgomery HE et al reported that the ACE genotype distribution and allele frequencies were similar in patients and control subjects. [7] Furthermore, current evidence supported the inhibition of renin-angiotensin system might be beneficial to patients of DCM. Similarly, the association between ACE I/D polymorphisms and HCM was also inconsistently reported [8], [9].

To date, no large-scale studies have assessed the association between ACE I/D polymorphisms and DCM or HCM. This lack of knowledge emphasizes the importance of the present meta-analysis. Thus, we performed this meta-analysis to clarify this inconsistency between ACE I/D polymorphisms and DCM or HCM.

## Materials and Methods

A computerized search of PubMed and the Cochrane Library published before November 2012 was conducted. Only studies published in English were considered. Furthermore, the references of the relevant studies were also searched. The google scholar website was also searched. When the same patient population was included in different reports, only the study with complete data was used in this meta-analysis. We used the following key words for searching for the relevant reports: angiotensin I converting enzyme, dilated cardiomyopathy, hypertrophic cardiomyopathy, variant and polymorphism. Two reviewers (J.M Yang and C Zhang) independently searched the titles, abstracts, and full-texts to determine whether the data met the inclusion criteria. Conflicts were resolved by consensus.

### Inclusion Criteria

The studies included in the meta-analysis must meet all the following three criteria: (1) evaluating the association of ACE I/D polymorphism with DCM or HCM; (2) using case-control design; (3) providing sufficient data upon genotype counts.

### Exclusion Criteria

All the patients were excluded for ischemic cardiomyopathy and severe coronary obstruction for DCM, and potential stimulus such as hypertension, ischemic ischemic heart disease, valvular heart disease, congenital malformations of the heart or vessels, and intrinsic pulmonary disease for HCM.

### Data Extraction

For each study, the following information was extracted: the first author’s name, publication date, region and ethnicity of participants, sample size of cases and controls, source of controls, myocardial biopsy, genotype distribution in cases and controls.

### Statistical Methods

All the statistical analyses were performed by Review Manager version 5.1. Crude odds ratios (ORs) and 95% confidence intervals (CIs) were used to assess the association strength between ACE I/D polymorphism and DCM or HCM risk. We also tested the heterogeneity among the included reports and *P*<0.10 was considered to be significant heterogeneity. In this study, a random effects model was used because of the presence of heterogeneity. Because the number of included studies was <10, we did not assess the publication bias (www.cochranehandbook.org).

## Results

### Characteristics of the Included Studies

**Figure 1:**
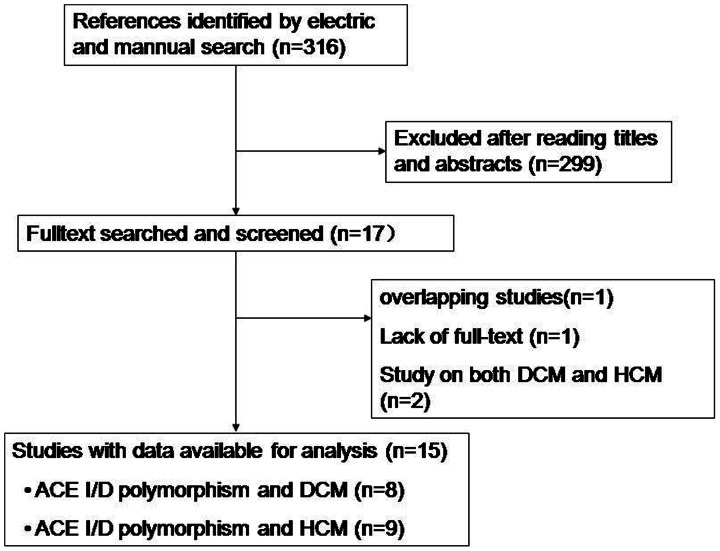
Flow chart of studies selection.

A total of 316 studies were screened and 299 studies were excluded after reading titles and abstracts. One study by Harn HJ et al. was excluded because of lacking full-text. For the two overlapping studies, [10], [11] the one published recently was included. [11] Two studies investigated the effect of ACE I/D polymorphisms on both DCM and HCM. [9], [12] Thus, eight studies on DCM (1387 controls and 977 patients, Table 1) and eight studies on HCM (1055 controls and 827 patients, Table 2) were included in this meta-analysis (Figure 1). Table 3 showed the genotype distribution in each included study.

**Table 1:** The characteristics of eligible studies on DCM considered in the meta-analysis.

Author	Year	Region	Ethnicity	Sample size (case/control)	Source of controls	Myocardialbiopsy	Diagnostic criteria
Raynolds MV et al. [6]	1993	USA	Caucasian	112/79	Healthy donors	Yes	EF<40%, LV enlargement and normal coronary arteries
Montgomery HE et al. [7]	1995	England	English	99/364	Healthy subjects	Yes	EF<40%, LV dilation and normal coronary arteries
Sanderson JE et al. [20]	1996	China	Chinese	100/100	Healthy subjects	Yes	EF<40%, fractional shortening<25% and with no ischemic cardiomyopathy
Yamada Y et al. [9]	1997	Japan	Japanese	88/122	Healthy subjects	Yes	DCM without other potential stimulus
Tiret L et al. [17]	2000	French	NA	422/387	Healthy subjects	No	EF<40%, LV dilation and with no ≥50% artery obstruction
Rai TS et al. [12]	2008	India	Indian	51/164	Healthy subjects	No	EF<40% and LV end diastolic diameter>117% of normal value
Kucukarabaci B et al. [21]	2008	Turkey	Turkish	29/20	Healthy subjects	No	NA
Mahjoub S et al. [16]	2010	Tunisia	Tunisian	76/151	Healthy subjects	No	LV fractional shortening <25%, EF<45% and LVEDD>69 mm

DCM, dilated cardiomyopathy; NA, not available; EF, ejection fraction; LV, left ventricular.

**Table 2:** The characteristics of eligible studies on HCM considered in the meta-analysis.

Author	Year	Region	Ethnicity	Sample size (case/control)	Source of controls	Myocardialbiopsy	Diagnostic criteria
Marian AJ et al. [22]	1993	USA	NA	100/106	Familiar healthy subjects	No	septal or ventricular thickness≥13 mm without other potential causes
Pfeufer A et al. [23]	1996	German	Caucasian	50/50	Healthy subjects	No	septal or ventricular thickness≥13 mm without hypertension and valvular heart disease
Yamada Y et al. [9]	1997	Japan	Japanese	71/122	Healthy subjects	Yes	LV hypertrophy without other potential causes
Ogimoto A et al. [24]	2002	Japan	Japanese	138/205	Healthy subjects	No	LV hypertrophy without other potential causes
Kawaguchi H et al. [11]	2003	Japan	Japanese	80/88	Familiar healthy subjects	No	LV hypertrophy without other potential causes
Rai TS et al. [12]	2008	India	Indian	118/164	Healthy subjects	No	Unexplained LV hypertrophy ≥13 mm or >2 standard deviations
Kaya CT et al. [8]	2010	Turkey	Turkish	63/20	Healthy subjects	No	LV hypertrophy ≥13 mm without other hypertrophic stimulus
Coto E et al. [19]	2010	Spain	Caucasian	207/300	Healthy subjects	No	LV hypertrophy ≥13 mm without other hypertrophic stimulus

HCM, hypertrophic cardiomyopathy; NA, not available; LV, left ventricular.

**Table 3:** The distribution of polymorphism for cases and controls.

			Genotypes (n)						Alleles (n)			
			Cases			Controls			I		D	
DCM			II	ID	DD	II	ID	DD	Case	Control	Case	Control
	Raynolds MV et al. [6]	1993	72		40	60		19	NA	NA	NA	NA
	Montgomery HE et al. [7]	1995	18	50	31	84	168	112	86	334	112	292
	Sanderson JE et al. [20]	1996	39	49	12	39	48	13	127	126	73	74
	Yamada Y et al. [9]	1997	36	35	17	50	55	17	107	155	69	89
	Tiret L et al. [17]	2000	94	200	128	71	190	126	388	332	456	442
	Rai TS et al. [12]	2008	8	33	10	47	87	30	49	181	53	147
	Kucukarabaci B et al. [21]	2008	5	18	6	7	9	4	28	23	30	17
	Mahjoub S et al. [16]	2010	12	38	26	46	83	22	62	175	90	127
HCM												
	Marian AJ et al. [22]	1993	7	49	44	22	46	38	63	90	137	122
	Pfeufer A et al. [23]	1996	26		24	36		14	NA	NA	NA	NA
	Yamada Y et al. [9]	1997	31	32	8	50	55	17	94	155	48	89
	Ogimoto A et al. [24]	2002	53	64	21	83	95	27	170	261	106	149
	Kawaguchi H et al. [11]	2003	26	41	13	43	28	17	93	114	67	62
	Rai TS et al. [12]	2008	11	63	44	47	87	30	85	181	151	147
	Kaya CT et al. [8]	2010	8	34	21	5	9	6	50	19	76	21
	Coto E et al. [19]	2010	35	100	72	46	135	119	170	227	242	373

DCM, dilated cardiomyopathy; HCM, hypertrophic cardiomyopathy; NA, not available.

### Association of ACE I/D Polymorphisms and DCM Susceptibility

Although the overall results demonstrated that the D allele carriers might be more susceptible to DCM, the risk did not reach a statistical significance. (DD/ID vs. II: OR = 1.34, 95% CI 0.92–1.95, *P* = 0.13; DD vs. ID/II: OR = 1.27, 95% CI 0.93–1.74, *P* = 0.13; DD vs. II: OR = 1.44, 95% CI 0.88–2.36, *P* = 0.14). Similarly, in the subgroup analysis by ethnicity, no significantly increased risk was found for D allele carriers among East Asians and Europeans. (Table 4).

**Table 4:** Summary of pooled ORs according to ACE I/D polymorphisms.

	DCM			HCM	
Comparison	Total	East Asian	European	Total	Japanese
Study (n)	8	2	3	8	3
**2/2 versus 1/1**					
OR(95% CI)	1.44(0.88–2.36)	1.16(0.64–2.11)	0.93(0.57–1.53)	1.67(0.90–3.11)	1.10(0.69–1.74)
*P* value for heterogeneity	0.01	0.51	0.18	0.0006	0.68
**2/2 versus 1/1+1/2**					
OR(95% CI)	1.27(0.93–1.74)	1.20(0.69–2.08)	1.02(0.81–1.29)	1.27(0.88–1.82)	0.96(0.63–1.48)
*P* value for heterogeneity	0.06	0.40	0.18	0.02	0.67
**2/2+1/2 versus 1/1**					
OR(95% CI)	1.34(0.92–1.95)	1.00(0.67–1.49)	0.91(0.68–1.22)	1.69(1.04–2.74)	1.20(0.88–1.63)
*P* value for heterogeneity	0.02	0.99	0.11	0.0009	0.17
**1/2 versus 1/1**					
OR(95% CI)	1.10(0.88–1.37)	0.95(0.62–1.45)	0.93(0.68–1.27)	1.62(1.06–2.46)	1.29(0.76–2.20)
*P* value for heterogeneity	0.12	0.74	0.12	0.01	0.09
**2/2 versus 1/2**					
OR(95% CI)	1.08(0.87–1.35)	1.22(0.68–2.20)	0.96(0.73–1.25)	0.99(0.79–1.26)	0.85(0.54–1.34)
*P* value for heterogeneity	0.20	0.36	0.90	0.26	0.36

ACE, angiotensin I converting enzyme; DCM, dilated cardiomyopathy; HCM, hypertrophic cardiomyopathy;

1/1, homozygosity for I allele; 1/2, heterozygosity; 2/2, homozygosity for D allele; CI, confidence interval; OR, odds ratio.

### Association of ACE I/D Polymorphisms and HCM Susceptibility

Overall, compared with ACE II genotype, patients with D allele showed a significant increased risk of HCM (DD/ID vs. II: OR = 1.69, 95% CI 1.04–2.74, *P* = 0.03). However, patients with DD genotype did not showed significant risk of HCM compared with II genotype (DD vs. II: OR = 1.67, 95% CI 0.90–3.11, *P* = 0.10) or I allele carriers (DD vs. ID/II: OR = 1.27, 95% CI 0.88–1.82, *P* = 0.20). In the subgroup analysis by ethnicity, no statistically increased risk was found for D allele carriers among Japanese. (Table 4).

## Discussion

To our best knowledge, our present study is the first meta-analysis to assess the association between ACE I/D polymorphism and DCM or HCM risk. Overall, our meta-analysis suggested that ACE I/D polymorphisms might be associated with HCM susceptibility but not DCM.

The angiotensin I converting enzyme enhances the synthesis of angiotensin II (Ang II), which induces cell proliferation, migration and hypertrophy, and enhances the proinflammatory cytokines and matrix metalloproteinases. Thus, overexpression of Ang II plays a powerful role in cardiomyopathy. Studies have demonstrated blocking Ang II is beneficial to patients with cardiomyopathy or heart failure. Previous studies have found that ACE I/D polymorphisms are related with plasma Ang II levels. ACE I/D polymorphisms have been extensively examined for a variety of clinical endpoints, such as hypertension, coronary artery disease [13], cough [14] and cancer [15]. The ACE I/D polymorphisms also modulate the phenotype in patients with DCM and HCM. However, studies from different populations have demonstrated conflicting data. Mahjoub S et al. found that DD genotype and D allele of angiotensin-converting enzyme I/D gene polymorphism are associated with increased risk of dilated cardiomyopathy in a Tunisian population, [16] while Tiret L et al did not find this correlation. [17] In the present meta-analysis, our overall results did not show significant association between ACE I/D polymorphisms and DCM risk. Furthermore, the subgroup analysis also did not find a statistically increased risk in D allele carriers among East Asians and Europeans, suggesting ACE I/D polymorphisms might not be associated with DCM risk. The previous data of ACE I/D polymorphisms in HCM patients also did not reach a consistency. Rai TS et al found that D allele of ACE I/D polymorphism significantly influences the HCM phenotypes. [12] However, Yamada Y et al reported that the ACE I/D polymorphisms are not related to HCM in a Japanese population. [9] Our current meta-analysis found that compared with ACE II genotype, patients with D allele showed a significant increased risk of HCM, suggesting ACE I/D polymorphisms might attribute to HCM risk.

Several limitations should be considered. First, some genetic defects were known in HCM or DCM. However, all the included studies did not present such information, which might affect the analysis results. Second, heterogeneity among the included studies may affect the interpretation of the results of the meta-analysis. Third, most of the sample sizes of the referenced studies are relatively small, which might weaken the meta-analysis results. Furthermore, lamin A/C mutations in DCM and sarcomeric mutations in HCM contribute a lot to cardiomyopathy risk. [18] The study by Coto E et al found a possible association between AT1R A1166C but not ACE I/D polymorphisms and HCM. [19] Unfortunately, little was known about the role of ACE I/D polymorphisms in lamin A/C and sarcomeric mutations risk. Further study is needed to confirm this association.

Overall, the present meta-analysis suggested that certain ACE I/D polymorphism might be associated with HCM but not DCM susceptibility. The findings of the current study may add benefit to risk stratification strategies in patients with HCM and may encourage further study focusing on the effect of ACE I/D polymorphisms on HCM and risk. These results also suggest a potential treatment approach by regulating RASS in HCM patients. However, given the small sample sizes in this meta-analysis, further large random case-control studies are needed for further confirmation.
